# Can Diet Change the Course of Cancer?

**DOI:** 10.1021/acscentsci.2c00896

**Published:** 2022-08-11

**Authors:** Alla Katsnelson

p53 is a protein with a reputation. It works as a classic tumor suppressor, regulating cell growth and killing cells that have gone rogue by dividing too vigorously.

But about 15 years ago, researchers reported that when cells are starved of glucose, the protein changes loyalties: rather than executing tumor cells, it helps them survive. A few years later, Karen Vousden, a cancer biologist now at the Francis Crick Institute, and her then postdoc Oliver Maddocks, set out to see whether nutrients taken in through food can influence the p53 metabolic pathway.

They found that in tumor cells that lack p53, cutting off the supply of the amino acids serine and glycine slowed the cells’ growth, while a control diet containing the nutrients kindled it. Serine and glycine are nonessential amino acids, meaning they do not need to be consumed because the body produces them from other molecules, so it was not clear that taking them out of the diet would have any effect. Yet to the researchers’ surprise, the experiment worked the same way in mice injected with these tumor cells and fed a diet lacking serine and glycine. “The core discovery was that changing the diet can slow tumor growth. Just removing the two amino acids could do that,” Maddocks says.

Researchers have long speculated that a person’s diet can affect cancer, but until recently, most evidence for that has come from studies that survey the diets of large groups and do not look at the underlying processes happening in the body. Maddocks and Vousden’s finding was the first to demonstrate a direct biochemical link between diet and cancer. Since then, scientists have begun to see more connections. A flurry of recent studies has begun to reveal how specific nutrients such as sugars, amino acids, and fats might fuel or curb the growth of particular tumors.

So far, studies in animals are promising, but only a handful of clinical trials have been launched to test food-based approaches in people. “It’s a pipe dream in my view to think that diet [alone] is going to cure cancer,” says Matthew Vander Heiden, a cancer biologist at the Massachusetts Institute of Technology. “But it can make a difference, and we need to do the studies to figure that out.”

Scientists studying the biochemical link between diet and cancer make a lot of caveats. The most important one: researchers cannot yet tell patients what to eat when they have cancer. Despite media headlines touting the cancer-fighting properties of blueberries or broccoli, “the truth is, right now we don’t have any recommendations,” says Naama Kanarek, a cancer metabolism researcher at Boston Children’s Hospital and Harvard Medical School. “This is very important to emphasize.”

**Figure:**
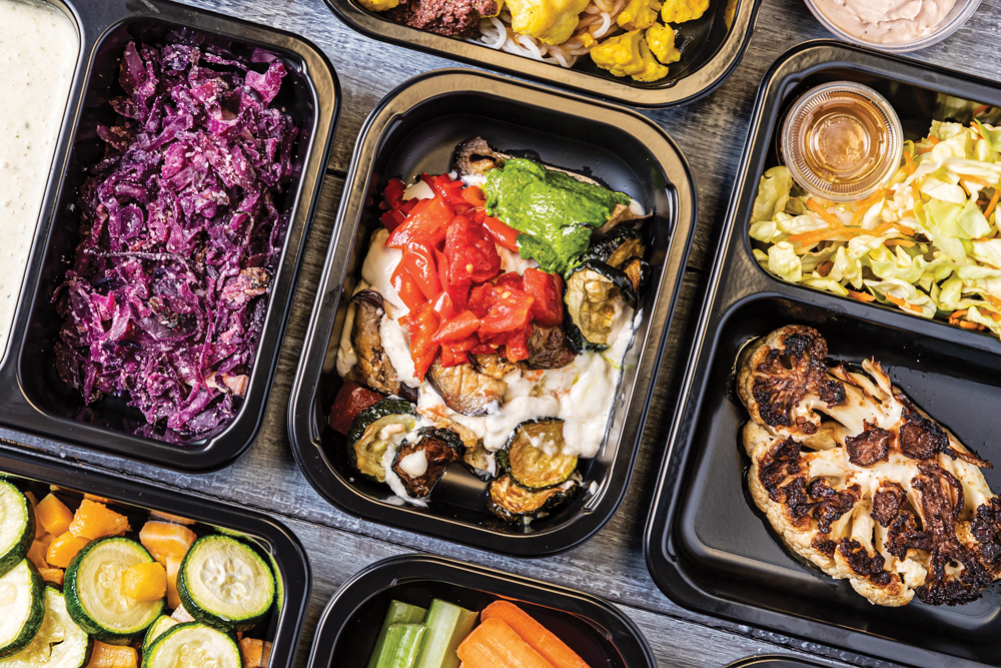
Faeth Therapeutics is testing whether a diet specially formulated by the company that is depleted in serine and glycine improves response to medicine in people with pancreatic cancer. Credit: Faeth Therapeutics.

There is almost certainly no single food or dietary fix that would help address all types of cancer, says Jason Locasale, a cancer researcher at Duke University who studies the role of amino acids and other nutrients in cancer. It is also highly unlikely that dietary interventions will completely replace cancer-killing medicines as therapies, he adds.

Instead, as researchers learn more about the underlying biochemistry, they envision creating a kind of matrix that would match cancer-causing genetic mutations or parts of metabolic pathways to precise nutritional approaches that complement cancer medicines. Many cancer drugs add just a few months to a patient’s life, and for unknown reasons, some people do not benefit from some medications at all, even if their tumor types predict they should. With the proper matchup, the right nutrient punch could boost cancer drugs’ efficacy and make more people responsive to them, Vander Heiden says.

The idea that nutrients can directly fuel tumor cells is not new. Almost a century ago, the German biochemist Otto Heinrich Warburg discovered that tumor cells gobble up an enormous amount of glucose. Warburg’s discovery laid the foundation for the field of cancer cell metabolism, and for decades, researchers investigated cancer through that lens.

Indeed, the very first potent cancer drugs were based on the effects of cell metabolism. In the late 1940s, while pediatric pathologist Sidney Farber was treating children with anemia—a condition brought about because of their leukemia—he found that using folate (vitamin B9) could make the leukemia advance surprisingly quickly. That led him to successfully treat their leukemia with methotrexate, a drug that inhibits folate activity. Methotrexate is still widely used against cancer and several other diseases today.

In the 1980s, though, cancer researchers shifted their focus away from metabolism and toward the genetic signatures of cancer. The search for small molecules that could drug these specific mutations yielded several key cancer therapies, but ironically, that research directed them back toward cell metabolism, explains Lewis C. Cantley, a biochemist at Dana-Farber Cancer Institute and Harvard Medical School. “Everyone was focused on oncogenes, and now we find out what they do,” he says. “They regulate metabolism, which gets us back to where we started.”

Research on an enzyme that Cantley discovered in the mid-1980s called phosphoinositide 3-kinase (PI3K) has helped power that shift. He and his colleagues found that PI3K is a glutton for glucose: given the right conditions, PI3K will fuel tumors’ growth as long as it has the sugar available. It is also mutated in an enormous variety of cancers. Several PI3K inhibitors have been approved to treat cancer, but they often have lackluster effects.

Today, Cantley thinks that previous studies may have overlooked the impact diet can have on PI3K inhibition and, specifically, that preventing insulin spikes may be one key to beefing up some cancer therapies. “We realized that insulin is by far the most potent activator of PI3 kinase,” he says. That means that drugs that shut down PI3K may not be enough to kill a tumor if a person’s blood insulin levels are too high. In a 2018 study, he and his colleagues proved this suspicion right. An insulin-lowering diet dialed up PI3K inhibitors’ efficacy, at least in mice.

Glucose may have an especially broad link to cancers because tumors feed on it, Cantley says. But there are many other nutrients to explore. Maddocks, now a cancer biologist at the University of Glasgow, is continuing to study tumor dependence on serine and glycine, looking beyond p53 to other aspects of cancer signaling that can hint at how serine and glycine depletion may be helpful.

Others are investigating the biochemical link between tumor cell proliferation and other amino acids, including aspargine, proline, leucine, and methionine. In 2019, Locasale of Duke and his colleagues reported that in mice, one commonly used cancer drug as well as radiation treatment were much more effective when the animals ate a methionine-restricted diet.

How cells process amino acids can point researchers to other metabolic processes, too. When Kanarek was a postdoc in David Sabatini’s lab at the Massachusetts Institute of Technology, she studied the effect of histidine on cancer, and this led her back to the decades-old story of methotrexate and folate. She found that excessive amounts of histidine made cancer cells more sensitive to methotrexate, likely because of the one-two punch of the drug blocking folate activity and the cells burning through their folate supply as they break down the histidine. Her lab is now investigating how exactly and in what tumor types folate affects cancer metabolism, and whether supplementing histidine in the diet could make this drug, and potentially similar ones in the pipeline, more effective.

Other laboratories are looking at additional nutrients, such as lipids, as well as diets more broadly. Multiple studies have reported that ketogenic diets, which strongly limit carbohydrates while including high levels of fats, and diets that restrict caloric intake overall may combat cancer cell growth, both in a dish and in mice. Researchers have surmised this might be because of the diets’ lower glucose and insulin levels, yet the two diets seem to work differently.

As a postdoc in Vander Heiden’s lab, Evan Lien investigated why that might be. Both diets do decrease how much glucose tumors can access, but they also tamp down levels of an enzyme that synthesizes fatty acids, Lien and Vander Heiden found. It turns out, though, that the two diets change this enzyme’s playing field in different ways, explains Lien, who now leads his own metabolic research lab at Van Andel Institute. The ketogenic diet is high in fats, so tumor cells can just use those ingested lipids rather than depend on the enzyme. But the caloric restriction diet tends to lower lipid levels overall, which may make tumors more dependent on the enzyme. So with that diet, cancer cells grow more slowly because they can neither synthesize fatty acids nor acquire them from their environment.

Ultimately, how any dietary intervention affects a tumor depends on the cancer’s genetic makeup, Lien explains. “Different tumor types with different genetic mutations can respond in distinct ways to the same diet, and we don’t totally understand why,” he says. A dietary intervention that curbs some tumors might make others proliferate better, so to match them, researchers still need to learn much more about the underlying biochemistry driving the metabolism of specific nutrients.

What’s more, translating dietary interventions from lab mice to people is tricky. It is easy to control what mice eat in a lab, but people may not be able or willing to sign on. Some people with cancer already struggle to maintain body weight, and others may have digestive or other issues limiting what they can eat because of their cancer.

In general, Kanarek says, diets that ask patients to eat something additional will probably be easier for people to handle than those that ask them to limit what they eat. Food is also a crucial component of emotional well-being, she adds, and stringent diets may have a mental health downside for some, though others might feel empowered by them. “I think it’s very important to incorporate psychological monitoring” in the studies, she says.

Some clinical trials have already grown out of the results of mouse studies. Three years ago, Cantley, Vousden, Maddocks, and others started a company called Faeth Therapeutics to test whether combining cancer drugs with foods could enhance the drugs’ response. The company’s name—*faeth* means nourishment and nutrition in Welsh—is a nod to food’s potential to boost the power of cancer medicines. Faeth is testing whether a ketogenic diet prepared for patients by the company will bolster the efficacy of two approved PI3K inhibitors, one made by Novartis and the other by Bayer, as well as a third that Faeth has licensed from Takeda. These trials are enrolling women with uterine and metastatic breast cancers that hijack the PI3K pathway.

Vousden and Maddocks are also leading a trial at Faeth testing a specially formulated diet depleted of serine and glycine that is given alongside standard treatment to people with pancreatic cancer, which proved to be especially dependent on these amino acids in mouse studies. Because different foods contain a variety of amino acid combinations, it would be difficult, if not impossible, to avoid just one or two amino acids by sidestepping certain foods. Instead, participants eat a diet engineered by Faeth that combines some “real” foods that are very low in protein—and therefore in amino acids—such as many fruits and vegetables, with a specially formulated shake that contains all the amino acids except for glycine and serine. “It is not something you can just go out and do on your own,” Maddocks says.

Other researchers, including Valter Longo, a cancer biologist at the University of Southern California and IFOM ETS–The AIRC Institute of Molecular Oncology, are enrolling patients in clinical trials investigating whether diets involving fasting, timed eating, and calorie and other nutrient restriction can boost the efficacy of medicines treating cancer.

**Figure:**
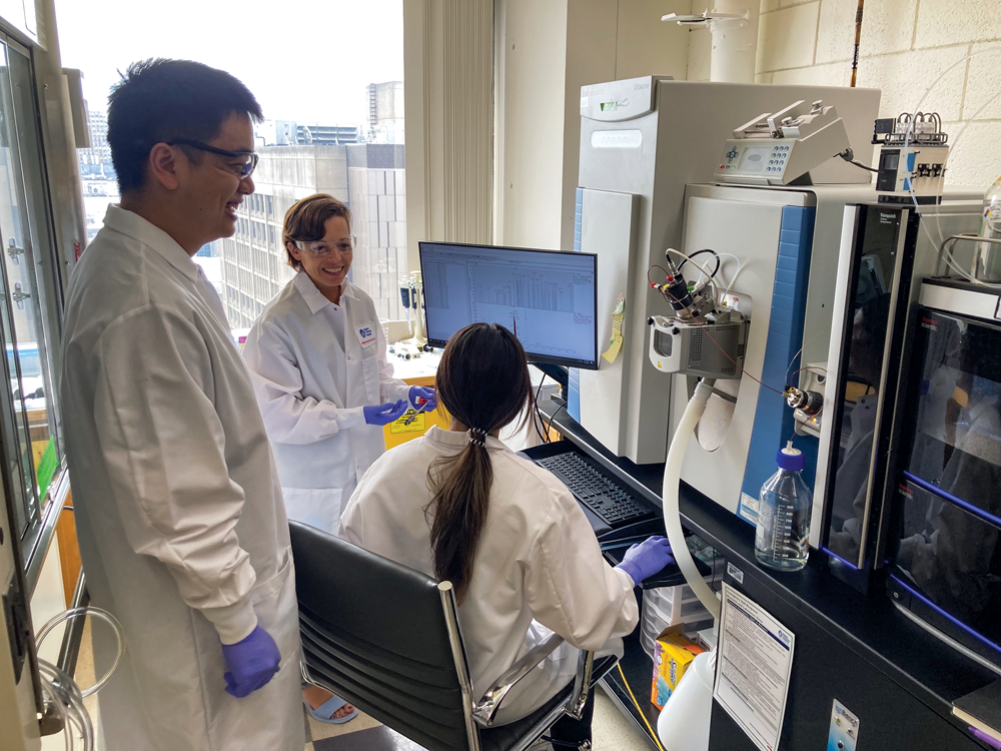
Naama Kanarek (center) and her colleagues are investigating folates effects on cancer metabolism. Credit: Adam G. Maynard.

Researchers say they have barely scratched the surface of the basic biology, considering that a vast number of nutrients could affect tumors’ biochemistry. “There’s an enormous number of interventions that could work,” Vousden says. “If the first clinical trial goes well and is positive, then I think enthusiasm for this approach will ramp up enormously.”

*Alla Katsnelson is a freelance contributor to**Chemical & Engineering News**, the weekly newsmagazine of the American Chemical Society.*

